# The Schools and the Teeth

**Published:** 1907-10-15

**Authors:** Charles O. Kimball


### THE SCHOOLS AND THE TEETH.

DR. CHARLES O. KIMBALL.

When some years ago I was urging upon one of my some what reluctant young patients the duty of personal care of his teeth and trying to fix in his mind the relation between eating and cleansing the teeth, and asking him to promise me to use brush and silk after breakfast each morning, I was met with the startling statement, for he was attending one of the best of New England's excellent schools: "I cannot do that for I am not allowed to go to my room after breakfast." So I retired, obliged to admit that his guns commanded my position.

It set me thinking, and asking other boys and girls, and wondering what I could do to make clear to the minds of the teachers that they were, as it seemed to me, failing in their duty. And when the notice of this meeting reached me and I was asked to prepare a brief paper, instantly it flashed into my mind that here was the opportunity I had been seeking. For we with our four societies fairly represent the best of dentistry in New England, and a reasonable section of it in New York, and if I can bring the force of your opinion and advice upon this subject fairly before the secondary schools, I am sure that no such answer could ever be given again from such a school.

This much by way of introduction. And now will you bear with me while I state as briefly as possible, and in untechnical terms, the position of modern dentistry on this subject, viz: the loss of teeth by decay and by their loosening.

Dental caries is fairly entitled to the bad pre-eminence of being the most universal of all diseases, as, to a greater or less degree, it exists in the vast majority of mouths and in most of the teeth in those mouths, and yet we are told that: "A clean tooth never decays." How can we reconcile these statements assuming them to be true. First by the definition of what a clean tooth really is—one that is kept entirely free from every trace of lodged foreign matter, a condition which upon a very brief thought is shown to be for some teeth almost impossible as regards, for instance, the minute fissures in the enamel, resulting from failure of union in development, which we know pass completely through it, and yet are too small to be completely cleansed without extirpation by filling or rounding out. I will therefore except these and place the responsibility otherwise. "A tooth if perfectly developed or if the fault of its development has been perfectly corrected by an absolutely sound filling, will not decay so long as it is kept clean.

But will such a tooth loosen and come out? I cannot give as yet the same confident answer, but there are those, and they are among the faithful ones, who "do the next thing" and do it well, who believe that the answer can be given in the same terms, that a clean tooth will remain firm in place to the end of life. I am not sure that this is true, but I know that the converse is, that an unclean tooth is in greater danger of loss by loosening than a clean one.

To keep teeth clean requires first regular, personal care, given at once; when the teeth are soiled; and second, regular professional care, given as often as may be necessary to supplement the personal care and keep the teeth up to the high standard of absolute cleanliness. This time varies with different individuals but the general failure of the profession in saving teeth is due to failing in these two particulars, to a lack on the part of the patient of thorough and regular personal care, and to a failure on the part of the dentist in cleansing the teeth with sufficient carefulness, regularity and frequency to supplement the personal care and so keep the teeth absolutely clean.

But as my object in this paper is not to quicken your zeal in your good work, but to reach those outside our profession, let me pass all this and ask, in a very simple elementary manner: What should be the character of the personal care of the teeth? How often? When? How? Where? By what means shall we clean our teeth? And to these come simple answers. How often? As often as they are soiled. When? Immediately after soiling them. How? By any means that shall be effective and yet not injurious to teeth or gums. Where? On every part of the surface of every tooth which can be reached by air or water. By what means? By a clean, moderately stiff bristle brush for all surfaces that it can reach, and by waxed floss silk for the surfaces the brush cannot reach. By these means and in this way we can keep our teeth clean.

To be ideal this care should begin as soon as the tooth appears above the gum, for if intermitted for a brief time decay is liable to cause a broken surface which will effectually neutralize all your efforts at this point. As the temporary tooth should receive the same care which its permanent supplanter receives, the parent or nurse must begin the work and by daily example, by careful teaching, and patient unwearied training so influence the young child that he shall form a habit of regular care. Thus the boy or girl grows until the first great break in life comes—going away from home to school. All recognize that this is a critical period, parental care and watchfulness are lost, what shall take their place, amid the confusing circumstances of the new life, who will see that the old habits are maintained? The teacher, who honestly and sincerely steps into the vacant place. When I think of the noble lives and high character of many of the men and women who have taken up this work in our secondary schools I see the reason for the success which they have in so many instances achieved in developing pure, high and true characters in their pupils.

But coming again to the care of the teeth, how are the boarding schools meeting this test of their wisdom and efficiency. They are training the mind, watching over morals, feeding the spiritual life, building up the physical life into vigorous strength, but how about the teeth, whose health will directly affect the physical life?

There are about one hundred and fifty secondary schools in Massachusetts and Connecticut; to each of these a circular was sent, asking five questions, conveniently arranged for a reply; answers were received from about seventy schools, some twenty of which proved to be day schools, and so out of the full scope of our inquiry.

To the first question, *Is there in this school any systematic effort to teach rules of hygiene of the teeth?* Of the day schools seven answered yes and fourteen no, boarding schools in about the same proportion. So that perhaps one-third of all the schools are giving, often in connection with their "Physiology" classes some instruction in the care of the teeth. But that is all. To the second question, *Is there any effort made to see that the pupils follow out such rules?* the response of the boarding schools alone was considered. Here the proportion was not quite so good, for thirty-six out of fifty over two-thirds did nothing. while four had advanced to the point of seeing that pupils cleaned their teeth "night and morning". Eight said yes unconditionally, and one said that pupils were sent to their rooms after every meal and then were watched to see that they cleaned their teeth. I would like to give the name of that school, for it seemed to me that it was doing all that it possibly could to give its pupils strong, sound teeth but I cannot, as all these answers were confidential. I found, however, one girls'

school in New Jersey where the pupils are required to clean teeth with brush and floss silk after each meal, and a trained nurse is by to see that they do it. When it came to the third question, *Is full opportunity given for the pupils to clean their teeth with tooth brush water and silk floss after each meal?* the proportion was reversed, for over two-thirds said they did, while nearly one-third said frankly "no," two of the best known schools taking the ground that the teeth were cleaned twice a day, morning and evening, as if they had in that fulfilled all the requirements of hygiene. The fourth question elicited no information of value while the last question *Is any care taken for the scholars' teeth?* brought out several interesting statements, for in nine schools there was more or less regular inspection of the pupils' teeth, in some, dental services were regularly employed to watch the children's teeth.

What does all this show? First that some schools are becoming interested, and are beginning to help, (a) by regular instruction, (b) by systematic watching, (c) and by planning so as to make care of the teeth by the pupils themselves easy and convenient. Second, that a greater number are quite indifferent as to instruction and still more as to endeavoring to train scholars in this particular, several of the most prominent only requiring care night and morning, but not allowing care after every meal except by special permission. Third, that many of the teachers themselves have a low standard of personal care, and so their training is ineffective, or by negation positively harmful.

Many dentists are now claiming that in the practice of orthodontia the years from six to twelve are perhaps the most important of all, as the developing jaws can then be made to develope on normal lines. Be that as it may, the years from twelve to twenty are propably the most critical in the history of the teeeth in their individual relation to disease. How many times our sympathies are excited by seeing the irremediable loss incurred during school life; not only complete loss of teeth by extraction, but still more frequently the irreparable loss of strength of a tooth by means of a great cavity, burrowed out in each approximal surface, or by fissure cavities enlarged till the enamel cap is left a mere shell. We can save them, O yes! by extensive upbuilding, by large and disfiguring fillings, but the beauty and the strength of the tooth are largely gone forever, and this may have occurred in two or three years time between twelve and twenty. I have seen it happen in about ten month's time. And this critical time of life of the teeth is the very time when the young creatures, boys and girls, whose welfare is so close to our hearts and consciences, are placed in the care of boarding schools.

Now, what can we do? How can this evil be remedied? Fortunately, we have at hand a powerful aid if we can invoke it, for the teachers of these young persons are men and women of high ideals, who have deeply at heart the true welfare of their charges, and they will surely respond if we can reach them with an appeal which has reason beneath it, and authority behind it.

Here is our present rare opportunity; four societies representing perhaps 600 dentists, and these pupils for whom I plead are our professional children, whom we have watched from babyhood.

Let us as a united and earnest body of men make a direct appeal to these teachers, asking them to do two things: First, to provide in their instruction for simple talks on the care of the teeth, the reason for it and how to do it, and then to arrange the mode of life of the scholars, so that it shall not only be possible for each boy and girl to cleanse his or her teeth after every meal and before retiring, but taking advantage of the discipline and routine of school life, that it shall be seen to that the scholars do give this personal care at these frequent stated times, and so supplement the home training by the school training, establishing a personal habit.

I have prepared a brief catechism of dental hygiene which I offer as a suggestion. If it meets with your approval

I should like to see it printed not over my name, but over yours, in the name of these four societies here in joint session.

I have also suggested an appeal to the schools which might be referred to a suitable committee, to revise or rewrite and send out, in such manner as they deem wise.

In conclusion, let me beg of you very careful consideration of the subject; it is a little matter, but it means much, extending over a long time. Who can tell how far our action this day may extend its influence, what blessing it may bring into the world, how far it may go towards helping to train up a stronger and better race of men and women?

If you can do this in the private schools you may expect it to extend to the public schools so that the teachers shall train their children to care for their teeth and so indirectly for their health. Booker Washington says that the first upward step is the use of a tooth brush.

### DISCUSSION.

Dr. Robert Whitehill: It seems to me we should not go from here without making this meeting count for something vital on the question.

Dr. Kimball has shown us the need of following the early care of the teeth in later years when the children have passed from our direct care. We have at hand the remedy if we can invoke it. The most significant fact of Dr. Kimball's paper is that dentistry is speaking to the world. We are asked to speak to the world.

The members of each profession are daily dealing with the public individual to individual. The medical profession not only does the work of routine which falls to the lot of medical practitioners, but the medical profession is reaching out all the time in an effort to promote the public health in hygiene, etc. In saving teeth we are daily doing our duty, but we must also reach out in an effort to educate and instruct the public, that we may secure intelligent co-operation in this worthy work.

Dr. Charles E. Parkhurst: We have all been very much interested and impressed by this presentation of Dr. Kimball, and are in harmony with its suggestions. That which struck me most forcibly in the paper was the need of universal adoption and application of these suggestions.

The need of emphasis in the care of the teeth at this class of schools is certainly apparent, as we have all borne witness.

The doctor kindly furnished me with a brief synopsis of this paper some days ago, but it was not until I saw the paper in full the day before yesterday that I was made aware that his remarks were relative to our secondary schools alone. I took the subject to include our public schools, which are so lacking in their attention to mouth cleanliness. With this understanding I had made my preparation and consequently offer this excuse for my remarks, which may not be quite appropos to the restricted subject, yet I believe that they would apply to all educational systems.

Dr. Kimball's plan of work in explaining and advising the frequent thorough cleansing of unclean teeth and the education of those to whom the children are intrusted is the practical way of accomplishing our purpose.

Oral hygiene in the public schools has been under discussion for some time.

In 1882-3 Dr. Parreidt, in Germany, and Dr. Ottofy, in this country, made the first examinations of students' teeth and tabulations thereon. In 1902-3 examinations were made in several states by certain individuals, and such have been made also in several European countries.

These tabulations, showing that 90 percent. of the poor children's teeth are defective in the United States, 75 percent. in England and 95 percent. in Germany acquaint us with the deplorable condition of our scholars' teeth the world over.

The conditions existing, what can be done to better them? The solution is not an easy problem. It is to take a long time to get the remedy into working order. *Medical inspec-*

*tion* in the schools is a slow process. It is uphill work to impress upon those in the community connected with the cause of education that more time should be devoted to the subject of Oral Hygiene.

Nevertheless, though the task does not progress rapidly, there is a trend in the world toward the betterment of hygienic conditions.

Pure food laws, the suggestion of our own *Governor* that steps be taken toward the improvement of the surroundings for the health of factory children with an inclination toward tuberculosis, experiments in various parts of our country regarding the special education of defective children, all these steps are making for the uplifting physically of our social and working communities. In each one of these there is a connecting link with our own line of work.

The system of education is such to-day that with the excessive mental development does it not seem rational, as is stated, that the teeth, jaws and face are suffering at the expense of the brain? Our school committees, perhaps being made up of those exemplyfying marked activity in business or education, and in many cases personally neglecting their own mouths, do not readily comprehend the need of much care of those organs coming under our control.

The entering wedge for the subject of Oral Hygiene in our schools may lie in revealing the fact that the unhealthy mouth of the scholar may be directly responsible for the spread of contagion and infection in the community.

So, gentlemen, in this matter it is upon us as intelligent, up-to-date dentists, conversant with all that is best in our work, that the responsibility rests, and it is our duty to see to it that everything is done which is possible.

We must be the educators of the teachers of the scholars, of the public and of the medical practitioners, if you please.

You can but have realized the lack of knowledge which our teachers possess relative to dental matters. Many of their mouths are a witness to this truth.

We must talk more in our offices and instruct when some diseased condition presents itself. Facts regarding the mouth and associated parts, together with the sequellae of these diseased organs, are in the province of the dentists to elucidate.

While caries is a very important aspect as regards the health of the mouth, other pathological states are more important. Upon this side of the subject is there great lack of knowledge.

Did you read the editorial in the September *Cosmos?* It was the most convincing short article upon this subject which I have seen. I have read and reread it and placed it on my reception room table for my patients. I would that the public might have access to it. We should know and impart to our patients, teachers and others the supreme importance of healthy mouths that Oral Hygiene may of necessity become a part of our children's training in school. The teachers are the instruments upon whom devolve the responsibility of the selection of suspicious pathological cases in school for our medical inspection, consequently their need of knowledge in this matter, likewise, also, their need of knowing the bearing the mouth and teeth may have upon the case in question.

Let the teachers and the public know that troubles with the eyes, one-fourth of all earache, throat and nose troubles, tuberculosis of the lungs, adenoids to which it is said 90 percent. of the children of New York are suffering and to which 50 percent. of all deafness is due, facial neuralgia, influenza, blunted mental activities, backwardness at school, diseases of the nervous system—that all these may be closely connected or originated from unhealthy mouths.

Let it be known that: There is a more or less marked ratio between the physical soundness and the mental acuteness of the child and the conditions of the teeth; that dental pain causes more suffering to the human family than any other disease.

Let it be known that extracting should never be resorted to except in positively unhealthy, unsalveable teeth. Let the importance of frequent daily cleansing on the part of the individual and periodical cleansing at the hands of the dentist in order to keep the teeth from decaying and the tissues healthy be told again and again.

Dr. Kimball has spoken of the dental primer. This is a splendid idea, and should have endorsement. School physiology should have a larger part devoted to this subject, and written by a dental practitioner and not by a practitioner of general medicine.

As is being done in the course of free lectures at the Harvard Medical School, Oral Hygiene as portrayed by one of our capable practitioners should have a place. Dr. Lavett, in his talks relative to the health of school children, says:

"It is a question whether defects of the eye and ear, of malnutrition, etc., are not as important as book learning, and if one has to be sacrificed, which should be the one? The chief objection is the conservatism of the community. We have, therefore, to be very patient, very conservative, ourselves, in hope for the day when people realize that the conditions under which their children go to school are as important as their drinking water and their plumbing."

These, gentlemen, are some of the thoughts which have come to me regarding the schools and the teeth. I have stated my reason for the digression from the subject, and yet be the school at our next door or in another city; be our children here or there, our aim in all events is the same; namely, to prevent decay and other oral disorders by emphasizing the means of avoiding them.

In the fulfilling of such a mission we can lay claim boldly to a place with general medicine in helping to bring about the well-rounded-out physical type of humanity, fitted to cope with the many struggles of life. I thank Dr. Kimball for his paper and for opening up this new field of work.

Dr. J. F. Dowsley: I feel that Dr. Kimball's paper should treat of the children of the masses rather than the children of the classes. It is these children whom we should deal with, for we shall have these for our future citizens. We have 100,000 children in attendance at our public schools and 8,000 in the private schools and in addition the parochial schools.

The private schools are usually supported by rich families. To show you that the things we are interested in already on the way, I will read you a few statistics. In 1894 Germany established a dental clinic as a feature of the public school system. In England 38,000 school children were examined and a very large percentage had defective teeth.

In Prussia the large majority were affected by caries. In one of our Massachusetts towns an examination was made, and 95 percent. of the children examined were affected with caries. I am well aware of the great work that is done by the colleges. I speak of Tufts and Harvard Colleges.

Here is a note relating to the Tufts Dental School:

The Tufts Dental School takes care of many thousands of the public school children's teeth in the course of the year, and I do not doubt that the Harvard School does the same thing.

In one of our public schools the children had to write a composition and the subject was: "What would you do with $50,000?" One would buy a piano, another would purchase a few frocks, a boy would immediately invest in an automobile; but there was one letter that interested the teacher from a little girl twelve years old.

The little girl twelve years of age said that if she had $50,000, the first thing she would do would be to have her teeth fixed.

That little girl is now receiving the best possible care at the Tufts Dental School.

A question discussed recently was: "How should the children be treated to insure a healthy mouth through life?" The concensus of opinion is to emphasize the importance of something being done in the way of training the children in this direction. Now, the teachers can do nothing because the schools are crowded. The child is sent home for dirty hands, but the clean mouth is not considered. Last year a committee was present in the Legislature considering the medical examination of children in the public schools.

That committee called in several gentlemen of high standing for their opinion. Among these several dentists were called in to get their opinions and ideas as to how the examination could be made. There was something in the air with regard to extending this to include a dental examination.

Everybody recognizes that this must come. And how we are going to accomplish that work is to get in some way in touch with the children of the public schools, because these children are going to be our future citizens. While it is fortunate for a child to attend a private school, yet the great majority are in the public schools. I hope these societies will make some expression that will reach the subject.—*The Journal.*

